# Multiple paxillin binding sites regulate FAK function

**DOI:** 10.1186/1750-2187-3-1

**Published:** 2008-01-02

**Authors:** Danielle M Scheswohl, Jessica R Harrell, Zenon Rajfur, Guanghua Gao, Sharon L Campbell, Michael D Schaller

**Affiliations:** 1 Department of Cell and Developmental Biology, University of North Carolina, Chapel Hill, North Carolina 27599, USA; 2 Department of Biochemistry and Biophysics, University of North Carolina, Chapel Hill, North Carolina 27599, USA; 3 Lineberger Comprehensive Cancer Center, University of North Carolina, Chapel Hill, North Carolina 27599, USA

## Abstract

**Background:**

FAK localization to focal adhesions is essential for its activation and function. Localization of FAK is mediated through the C-terminal focal adhesion targeting (FAT) domain. Recent structural analyses have revealed two paxillin-binding sites in the FAT domain of FAK. To define the role of paxillin binding to each site on FAK, point mutations have been engineered to specifically disrupt paxillin binding to each docking site on the FAT domain of FAK individually or in combination.

**Results:**

These mutants have been characterized and reveal an important role for paxillin binding in FAK subcellular localization and signaling. One paxillin-binding site (comprised of α-helices 1 and 4 of the FAT domain) plays a more prominent role in localization than the other. Mutation of either paxillin-binding site has similar effects on FAK activation and downstream signaling. However, the sites aren't strictly redundant as each mutant exhibits phosphorylation/signaling defects distinct from wild type FAK and a mutant completely defective for paxillin binding.

**Conclusion:**

The studies demonstrate that the two paxillin-binding sites of FAK are not redundant and that both sites are required for FAK function.

## Background

Focal adhesion kinase is a non-receptor tyrosine kinase that plays an important role in mediating essential cellular processes, such as cell growth, survival, and migration. FAK knockout mice exhibit embryonic lethality with defects in mesoderm, notochord, somites and the heart [1]. In addition, these embryos exhibit shortened anterior-posterior axes and vascular defects. Similar phenotypes have been described for fibronectin null mice [2] Paxillin null mice also share some of the phenotypes observed in *fak*^-/- ^embryos including truncated anterior-posterior axes and developmental defects in the heart and somites [3]. In accordance with the phenotypes of these mice, FAK plays an important role in mediating integrin signaling following cell adhesion to extracellular matrix proteins, e.g. fibronectin, and paxillin is an important physiological binding partner of FAK [4,5].

When integrins bind to the extracellular matrix they cluster on the cell surface resulting in the assembly of protein complexes, which include FAK, at these sites of adhesion. Cell adhesion induces FAK autophosphorylation at Y397, which creates docking sites for proteins with SH2 domains. The major binding partners recruited into complex with FAK following Y397 phosphorylation include Src, PI3 kinase, Grb7, and phospholipase C-γ [4]. Src subsequently phosphorylates other tyrosine residues in FAK. Y576 and Y577 are located within the activation loop of FAK and phosphorylation of these sites by Src regulates catalytic activity [6]. Src can also phosphorylate Y861 and Y925 in the C-terminus of FAK [7,8]. Phosphorylation of Y861 is induced by VEGF stimulation and promotes the formation of a complex between FAK and the αvβ5 integrin in endothelial cells [9]. Phosphorylation of Y925 may promote dissociation of FAK from focal adhesions [10]. By creating a binding site for the SH2 domain of Grb2, Y925 phosphorylation may be important for activation of ERK [8]. Grb2 binding to Y925 also recruits dynamin to focal adhesions, which is important for focal adhesion disassembly [11]. Thus, these Src-dependent phosphorylation events play an important role in regulation of FAK signaling downstream of integrin attachment.

The recruitment of FAK to focal adhesions is essential for its regulation by integrin-dependent adhesion to the extracellular matrix [12]. The C-terminal focal adhesion targeting (FAT) domain is responsible for the discrete localization of FAK to focal adhesions and this domain binds to two focal adhesion associated proteins, paxillin and talin [13-15]. Both of these proteins have been proposed to function in the localization of FAK to focal adhesions. Paxillin is a scaffolding protein containing multiple domains that mediate protein-protein interactions, including five N-terminal LD motifs, four C-terminal LIM domains, and SH2 and SH3 domain binding sites [16]. The second (LD2) and fourth LD motifs (LD4) of paxillin have been identified as FAK-binding sites and each of these sites binds to FAK with similar affinity [17,18]. *Paxillin*^-/- ^cells exhibit reduced localization of FAK to focal adhesions and reduced phosphorylation of FAK [3,19]. These results suggest that paxillin binding may function in the regulation of FAK activity, in addition to its proposed role in regulating localization.

Recent reports have used both NMR and crystallographic approaches to determine that the structure of the C-terminal FAT domain of FAK is a four-helix bundle [20-23]. Additional studies have also revealed the structure of the FAT domain of FAK in complex with peptides mimicking the LD2 peptide of paxillin [24,25]. The striking finding from these studies was the identification of two paxillin-binding sites on the FAT domain of FAK. This finding is particularly intriguing, given the presence of two FAK-binding sites in the N-terminus of paxillin. The two paxillin-binding sites in the FAT domain are structurally similar and consist of a surface exposed hydrophobic patch, adjacent to a series of basic residues. One paxillin-binding site lies at the interface of α-helices 2/3 and the other at the interface of α-helices 1/4 and thus the two sites are on opposite sides of the four-helix bundle. While evidence suggests that the LD2 peptide of paxillin interacts with both of these sites with similar affinity [24], the results of a paramagnetic labeling experiment suggests that the paxillin LD4 peptide has a preference for one site over the other [25].

These studies raise an interesting question of whether the two paxillin-binding sites of FAK are simply redundant interaction surfaces that strengthen the association between these two proteins, or alternatively, whether paxillin binding to each site might mediate a distinct function. To address these important questions, site-directed mutagenesis was used to disrupt binding to the α-helix 2/3 paxillin-binding site (E949A/K956A/R963A or EKR) or to the α-helix 1/4 paxillin-binding site (I937A). Combining these mutations together (E949A/K956A/R963A/I937A or EKR/I937A) completely abolished paxillin binding as demonstrated *in vitro *by GST pulldown and *in vivo *by co-immunoprecipitation as previously reported [24]. These mutants have been characterized to determine the role of paxillin binding to each site individually in the regulation of FAK.

## Results

**Figure 1:**
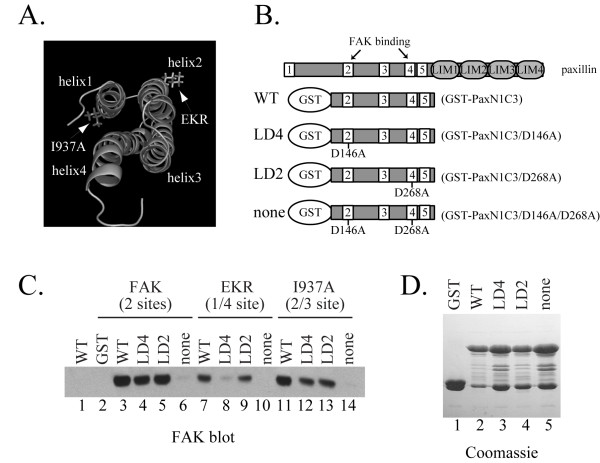
***In vitro *binding of paxillin and FAK mutants**. **A) **The I937A and EKR mutations are indicated on the structure of the FAT domain. The four α-helices are labeled. The paxillin binding sites are at the interface of α-helices 1/4 and the interface of α-helices 2/3. **B) **Schematic representation of paxillin and paxillin GST-fusion proteins used for the binding studies. White boxes denote the LD motifs. The proteins are designated WT, LD4, LD2 and none to reflect functional LD motifs in the constructs. The previously published designation of each construct is in parentheses. **C) **CE cells transfected with empty RCAS vector (lane 1), wild type FAK (lanes 2–6), EKR (lanes 7–10), or I937A (lanes 11–14) were lysed and 1 mg of lysate was used for binding assays. The functional paxillin binding site in each of the constructs is indicated in parentheses. Lysates were incubated with GST alone (lane 2), the WT (lanes 3, 7, 11), the LD4 (lanes 4, 8, 12), the LD2 fusion proteins (lanes 5, 9, 13), or with the protein lacking both LD2 and LD4 sites (none; lanes 6, 10, 14). The protein complexes were washed and bound FAK detected by Western blotting. **D) **GST-fusion proteins used in **C **were analyzed in parallel by SDS-PAGE and Coomassie staining to ensure equal loading.

Paxillin has two FAK binding sites and mutants of paxillin that disrupt binding to FAK have been previously described [18]. Recent studies have revealed that FAK also has two binding sites for paxillin. The I937A mutation is in helix 1 and destroys paxillin binding to the helix 1/4 binding site but retains paxillin binding to the helix 2/3 binding site (Fig. 1A)[24]. Conversely, the EKR mutations are in helix 2 and abrogates binding to the helix 2/3 binding site, yet retains binding activity through the helix 1/4 binding site (Fig. 1A)[24]. To assess the ability of individual binding sites on paxillin and FAK to interact, a GST pulldown approach was utilized. GST fusion proteins, shown in Figure 1B, were used to bind to FAK variants expressed in CE cells. The GST fusion proteins contained the N-terminal portion of paxillin containing both FAK-binding sites, i.e. LD 2 and LD 4 (GST-PaxN1C3). GST fusion proteins with point mutations that disrupt FAK binding to LD2 (D146A) or LD4 (D268A) were also used, as well as a double mutant that abolishes FAK binding to both LD2 and LD4 (D146A/D268A). Thus, these fusion proteins contained a functional LD4 motif, a functional LD2 motif and defective LD2/LD4 motifs respectively. Fusion proteins, immobilized on glutathione beads, were incubated with lysates of CE cells expressing FAK variants and bound FAK assessed by western blotting. Wild type FAK bound to the wild type recombinant paxillin, exhibited slightly reduced binding to recombinant paxillin with one functional LD2 or LD4 motif, and bound poorly to the double mutant (Fig. 1C, lanes 3–6). These observations are similar to published results using these paxillin mutants [18]. The same trend is observed with the I937A FAK mutant, i.e. reduced binding to the LD2 and LD4 mutants and poor binding to the double mutant (Fig. 1C, lanes 11–14). This result suggests that the I937A mutant (containing the intact helix 2/3 paxillin-binding site) can bind to both the LD2 and LD4 FAK-binding sites of paxillin. In contrast, the EKR FAK mutant exhibits a different pattern of binding. This FAK mutant binds the wild type paxillin fusion protein and shows reduced binding to the paxillin mutant with a functional LD2 motif (Fig. 1C, lanes 7 and 9). The EKR mutant binds very poorly to the paxillin mutant with a functional LD4 motif and the double mutant (Fig. 1C, lanes 8 and 10). This result suggests that the EKR mutant (containing the intact helix 1/4 paxillin-binding site) binds to the LD2 FAK-binding site of paxillin, but binds very poorly to the LD4 FAK-binding site of paxillin. These results reveal that the two paxillin-binding sites of FAK do not interact with paxillin in precisely the same way. Both paxillin-binding sites of FAK appear to bind to the LD2 motif of paxillin (Fig. 1C, lanes 9 and 13). However, the helix 2/3 binding site binds the LD4 motif of paxillin (Fig. 1C, lane 12), while the helix 1/4 binding site is defective (Fig. 1C, lane 8). Thus the LD4 motif preferentially associates with the interface of α-helices 2 and 3, whereas the LD2 motif can associate with either paxillin-binding site.

**Figure 2:**
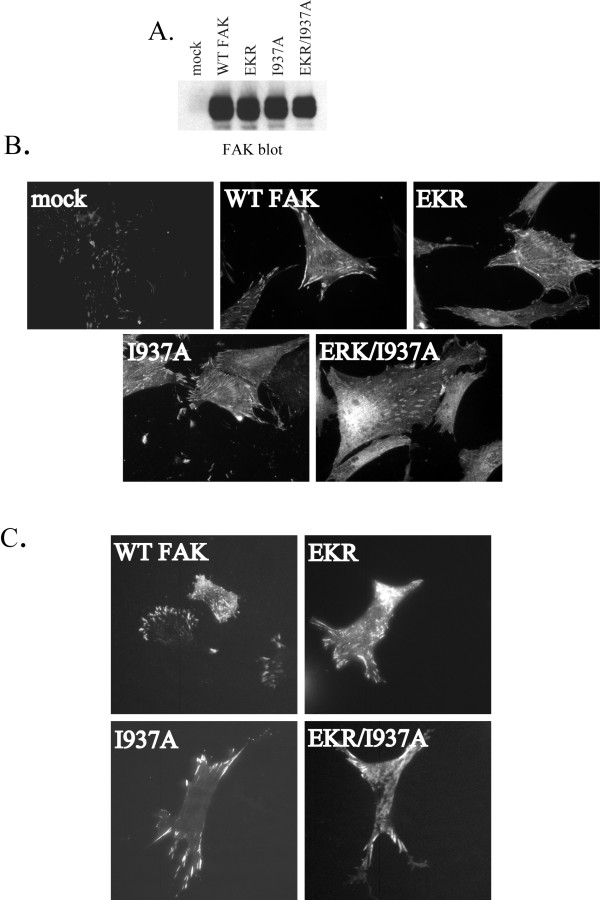
**Subcellular localization of FAK mutants**. **A) **CE cells transfected with empty RCAS vector (mock) or RCAS constructs encoding wild type FAK or FAK mutants were lysed and 25 μg of lysate was blotted for FAK expression with BC4 antibody. **B) **CE cells transfected with empty RCAS vector (mock) or RCAS encoding wild type FAK or FAK mutants were plated on fibronectin-coated coverslips overnight, then fixed and used for immunofluorescent imaging using the FAK BC4 antibody. **C) **CE cells transfected with GFP-fusion constructs of wild type FAK or mutants were visualized in live cells by TIRF microscopy.

**Figure 3:**
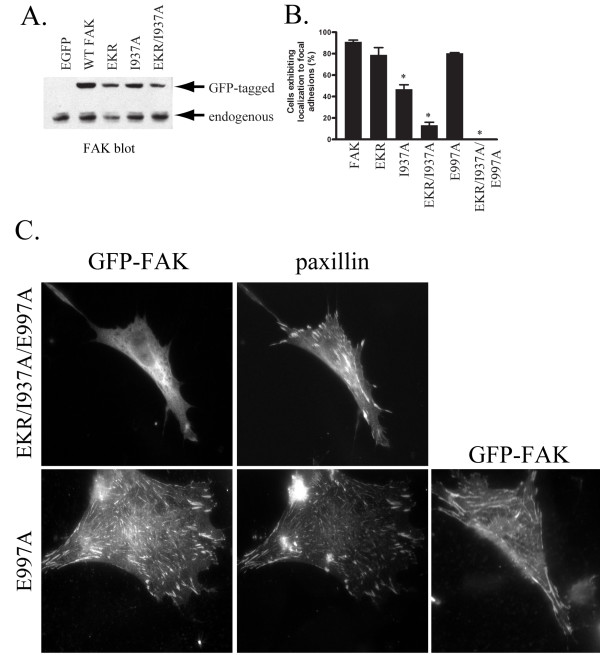
**Quantification of focal adhesion localization**. **A) **CHO-K1 cells were transfected with the empty EGFP vector or GFP N-terminally tagged versions of wild type or FAK mutants. Stably expressing populations were selected and lysed. Twenty-five μg of lysate was immunoblotted to examine FAK expression. Endogenous FAK and GFP-FAK are indicated. **B) **CHO-K1 cells stably expressing the EGFP vector alone, or GFP-FAK fusion proteins were plated on fibronectin coated coverslips overnight, then fixed and used for immunofluorescent staining with a GFP antibody. The cells were counterstained with a paxillin antibody to locate focal adhesions. GFP-positive cells were scored for GFP localization at focal adhesions. The data was analyzed using a one-way analysis of variance and the Dunnett post test (* denotes p < 0.01). **C) **CE cells transfected with GFP-FAK fusion proteins were plated on fibronectin-coated coverslips overnight, then fixed and used for immunofluorescent staining with a GFP antibody. The cells were also stained with a paxillin antibody to facilitate identification of focal adhesions. A second example of GFP-E997A localized to focal adhesion is also shown (lower right panel).

The subcellular localization of FAT domain mutants was assessed by immunofluorescence of exogenously expressed protein. Initially, CE cells expressing exogenous FAK using the RCAS retroviral vector were analyzed. Cells were plated onto fibronectin-coated coverslips and allowed to adhere overnight. Coverslips were fixed, permeabilized, and stained with a FAK polyclonal antibody and FITC-anti-rabbit secondary antibody. The exogenous proteins are expressed at high levels making it easy to distinguish their expression (Fig. 2A). Examples of cells exhibiting focal adhesion localization of each of the FAK mutants are shown in Figure 2B. To validate these findings, a complimentary approach using GFP-tagged FAK variants was used to assess localization. GFP fused to wild type FAK or the FAK mutants were transiently expressed in CE cells. At 24 hrs post-transfection, the cells were plated onto glass-bottom dishes and 24 hours later, the cells were viewed by epifluorescence and TIRF microscopy. Each of the FAT domain mutants exhibited focal adhesion localization by both epifluorescence (data not shown) and TIRF microscopy (Fig. 2C). These experiments revealed that mutants of FAK that disrupt paxillin binding to either site on the FAT domain or to both sites can still localize to focal adhesions. However, during the course of imaging the transfected CE cells it was apparent that some of the mutants were more difficult to find at focal adhesions, suggesting there might be a quantitative difference in efficiency of localization. To further investigate the subcellular localization of these mutants, stable populations of CHO cells expressing GFP-FAK fusion proteins at a similar level as endogenous FAK protein were established (Fig. 3A). Cells were plated on glass coverslips and allowed to spread overnight before being fixed and stained using an avian GFP antibody. Focal adhesions were stained using a paxillin monoclonal antibody and a rhodamine-labeled secondary antibody. Fixed cells were imaged and paxillin staining was used to identify focal adhesions. Cells were scored for co-localization of paxillin and GFP staining, indicating FAK localization at these sites. The results were expressed as percentage of cells exhibiting focal adhesion localization of GFP-FAK (Fig. 3B). Like wild type FAK, the EKR FAK mutant was targeted to focal adhesions in about 90% of the cells. The I937A mutant was localized to focal adhesions in about 55% of the cells, indicating that this paxillin-binding site may be more important for focal adhesion targeting than the other paxillin-binding site. Although some cells expressing the mutant EKR/I937A, which has defects in both paxillin-binding sites, exhibit focal adhesion localization of the FAK mutant, the efficiency of targeting was severely decreased compared to wild type FAK. These results indicate that although paxillin binding is not absolutely required for FAK localization, this association does play an important role in FAK's ability to localize to focal adhesions.

Although the above experiments indicated that paxillin plays a major role in the regulation of FAK targeting to focal adhesions, the FAK mutant defective for paxillin binding still correctly localized in approximately 10% of cells. This mutant presented a suitable background for additional mutagenesis studies to identify residues outside of the paxillin-binding regions of the FAT domain that are important for FAK localization. Additional alanine substitutions at conserved residues on the surface of the FAT domain were made and the subcellular localization of the resulting mutants was assessed upon transient expression as GFP fusion proteins in CE cells. Using this approach, E997 was identified as a residue important for FAK localization to focal adhesions in the absence of paxillin binding (Fig. 3C). This mutant was further analyzed by stable expression of the GFP fusion protein in CHO cells. The E997A mutation in combination with the EKR/I937A mutations resulted in a complete absence of the FAK mutant from focal adhesions. However, the E997A mutant in an otherwise wild type background, i.e. with intact paxillin-binding sites, was able to localize to focal adhesions nearly as well as wild type FAK (Fig. 3B and 3C). This result indicates that E997 is important for the localization of FAK to focal adhesions in the absence of paxillin binding.

**Figure 4:**
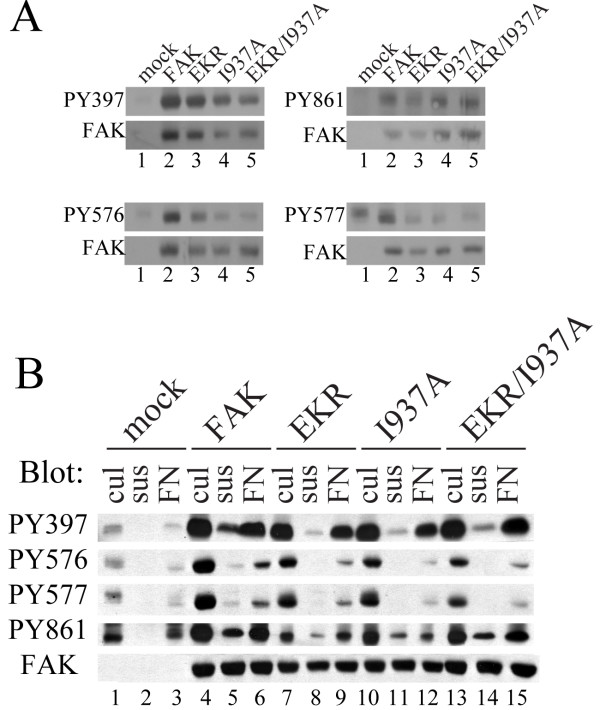
**Identification of defects in FAK phosphorylation**. **A) **CE cells were transfected with the RCAS empty vector (mock) or RCAS encoding wild type FAK or FAK mutants. Subconfluent cells growing in culture were lysed and immunoblotted with the indicated phospho-specific antibodies. In parallel lysates were blotted for FAK expression as a loading control. **B) **Phosphorylation of FAK variants was examined in cells growing in culture (cul)(lanes 1, 4, 7, 10, 13), after incubation in suspension for 30 minutes (sus)(lanes 2, 5, 8, 11, 14), and after plating on fibronectin for 45 minutes (FN)(lanes 3, 6, 9, 12, 15). Twenty-five μg of lysate was analyzed by immunoblotting using phospho-specific antibodies. Lysates were also blotted for FAK as a loading control.

As FAK phosphorylation is important for its activation and downstream signaling, and defects in FAK phosphorylation were observed in paxillin null cells [3], the mutants were characterized for phosphorylation defects. Cell lysates were probed with phospho-specific antibodies by Western blotting to assess the phosphorylation status of different sites in FAK in CE cells growing in culture. At the exposure shown in Fig. 4A, phosphorylation of endogenous FAK was barely detectable. The results indicate that Y397 is phosphorylated comparably on wild type and mutant FAK proteins (Fig. 4A). Since this site is the major autophosphorylation site of FAK, this experiment indicates that paxillin binding is not required for FAK autophosphorylation. Phosphorylation of Y861, like that of Y397, is not dependent upon paxillin binding as mutants that disrupt binding to individual sites or both sites have no effect on phosphorylation of Y861. In contrast, phosphorylation of Y576 and Y577 is dependent upon paxillin binding. Both of these sites in the activation loop of the kinase show reduced phosphorylation when paxillin binding is disrupted (Fig. 4A). Mutants with a single functional paxillin-binding site and the mutant lacking functional paxillin binding sites exhibited similar reductions in phosphorylation at these sites. Phosphorylation of FAK was also measured from cells stimulated by cell adhesion to fibronectin using phospho-specific antibodies. Note that at these exposures the phosphorylation of endogenous FAK is detectable, despite the inability to see endogenous FAK protein in the blot (Fig. 4B, lanes 1 and 3). This appears to be due to a higher stoichiometry of phosphorylation of the endogenous protein than exogenously expressed protein in this system (MDS, unpublished observation). The results indicate that Y397 and Y861 are phosphorylated in each of the mutants comparably to wild type FAK following cell adhesion (Fig. 4B). Phosphorylation of Y576 and Y577 is dependent upon paxillin binding following cell adhesion since phosphorylation was reduced in mutants that disrupt paxillin binding to at least a single paxillin-binding site in FAK.

**Figure 5:**
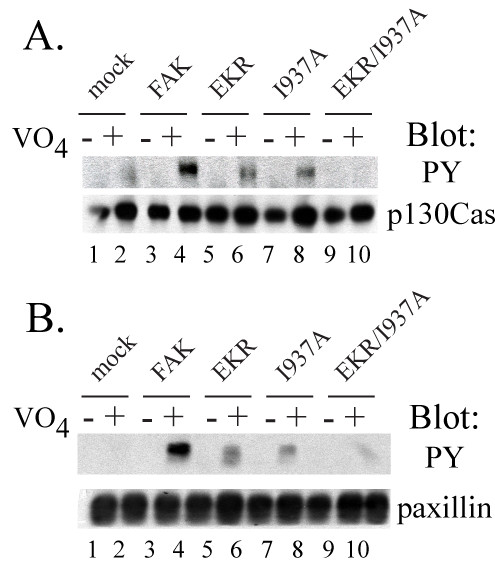
**Phosphorylation of FAK substrates *in vivo***. CE cells transfected with empty RCAS vector (mock) or RCAS encoding wild type FAK or FAK mutants were left untreated (-) or treated with vanadate overnight prior to lysis (+). **A) **p130CAS was immunoprecipitated from 1 mg lysate and the immunoprecipitated protein was immunoblotted for phosphotyrosine (upper panel) and p130CAS (bottom panel). **B) **Paxillin was immunoprecipitated from 1 mg lysate and the immunoprecipitated protein was immunoblotted for phosphotyrosine (top panel) and paxillin (bottom panel).

To assess downstream signaling of the various FAK FAT domain mutants, phosphorylation of FAK substrates was measured as previously described [26]. Briefly, CE cells expressing wild type or mutant forms of FAK were vanadate treated to prevent dephosphorylation of cellular proteins. FAK substrates p130CAS and paxillin were immunoprecipitated from cell lysates. Immunoprecipitated proteins were immunoblotted with a phosphotyrosine antibody, as shown in Figure 5. FAK mutants with a single paxillin-binding site induced tyrosine phosphorylation of p130CAS (Fig. 5A) and paxillin (Fig. 5B), although the levels of phosphorylation were reduced compared to the levels seen upon expression of wild type FAK. The FAK mutant with mutations at both paxillin-binding sites failed to induce phosphorylation of p130CAS or paxillin. These results indicate a role for paxillin binding in the transmission of signals downstream of FAK.

## Discussion

Recent structural analyses have revealed that FAK has two binding sites for paxillin. This raises the question of whether these binding sites are simply redundant, whether they act in concert, or whether engagement of distinct binding sites results in different outcomes. Site directed mutagenesis was used to disrupt paxillin binding to each individual binding site or to both binding sites simultaneously on the FAK FAT domain to begin to address these issues. Efficient localization of FAK to focal adhesions could occur with a single functional paxillin-binding site, although one binding site appeared more important for localization than the other. Both paxillin-binding sites appeared to work in concert to promote maximal phosphorylation of activation site tyrosines in FAK. Both binding sites were also required for maximal phosphorylation of downstream substrates, although a single binding site was sufficient to promote sub-optimal substrate phosphorylation.

A report measuring NMR chemical shifts upon titration of the FAT domain with synthetic paxillin peptides and paramagnetic labeling experiments suggested that the LD2 and LD4 motifs of paxillin bound differently [25]. The LD4 motif exhibited a preference for the paxillin-binding site between α-helices 2/3, while the LD2 motif exhibited a preference for the other binding site between α-helices 1/4 [25]. Using recombinant fragments of paxillin, with mutations impairing one or the other FAK-binding site, a similar conclusion regarding LD4 motif binding was drawn here, i.e. that the LD4 motif exhibits a preference for binding to the α-helices 2/3 side of the FAT domain. However, the results regarding LD2 motif binding are discordant. The results present herein demonstrate that the LD2 motif of recombinant paxillin binds comparably to both paxillin binding sites of the FAT domain, which is consistent with other published results examining LD2 motif binding to the FAT domain. ITC analysis determined that the binding affinities of the two paxillin-binding sites on the FAT domain for a peptide mimicking the LD2 motif of paxillin are similar, approximately 10 micromolar [24]. Further, ITC data suggested that the two binding sites were independent, as the binding isotherms for the wild type FAT domain binding a paxillin-derived LD2 peptide could be fit well to a two-independent binding site model. Consistent with this analysis, the I937A mutation abolished binding of the LD2 peptide to one paxillin-binding site and the EKR mutation abolished binding of the LD2 peptide to the other paxillin-binding site. It was therefore anticipated that the two binding sites would contribute equally to binding of full-length paxillin. However, binding studies using recombinant GST-paxillin fusion proteins and co-immunoprecipitation experiments demonstrated substantially more paxillin binding to the I937A mutant than to the EKR mutant [24]. Thus, the integrity of the α-helices 2/3 paxillin-binding site of the FAT domain of FAK is more important for the interaction with full-length paxillin than the other binding site. This observation is consistent with the hypothesis that the α-helices 2/3 binding site can interact with both the LD2 and LD4 motifs of paxillin, whereas the α-helices 1/4 binding site associates preferentially with the LD2 motif.

Although paxillin binding is not absolutely required for FAK subcellular localization, it is the major mechanism of focal adhesion localization. When paxillin binding is abolished, the FAK mutant localizes to focal adhesions with about 10% efficiency relative to wild type FAK. Further, the mutants disrupting individual paxillin binding sites differed in their efficiency of localization. The EKR mutant localized like wild type FAK, whereas the I937A mutant was partially defective. This seems surprising considering that the EKR mutant exhibits reduced binding to paxillin in an *in vitro *binding assay or by co-immunoprecipitation compared with the I937A mutant [24]. This indicates that paxillin binding does not precisely correlate with focal adhesion localization. For subcellular localization of FAK, the paxillin-binding site comprised of α-helices 1/4 is more important than paxillin-binding site comprised of α-helices 2/3.

Glutamic acid 997 was identified as an important residue for localization in the absence of paxillin binding. Note that this residue is on the α-helices 3/4 face of the FAT domain and lies outside of the two LD motif-binding sites. In the background of a FAK mutant that is defective for paxillin binding, mutation of this residue to alanine completely abolished focal adhesion localization, but in a wild type FAK background, this mutation does not significantly affect focal adhesion localization. Binding to this residue may represent a secondary, less efficient mechanism by which FAK can be localized to focal adhesions in the absence of paxillin binding. Talin is a focal adhesion protein that has been reported to bind to the FAT domain of FAK and has been suggested to function as a mechanism for FAK subcellular localization [15,27]. It is possible that E997 is an important residue for the association between FAK and talin. However, we have been unable to test this hypothesis, as we have been unable to detect the interaction between the FAT domain of FAK and talin by co-immunoprecipitation, GST pulldown or direct binding between purified recombinant proteins. Alternatively, E997 might be important for interaction with another binding partner and the E997A mutant might be a useful tool for the identification of additional physiologically relevant binding partners.

Phosphorylation of FAK is important for association with binding partners and for maximal catalytic activity. Results from paxillin null cells suggest paxillin binding is essential for maximal FAK phosphorylation [3,19]. The paxillin-binding defective FAK mutants have provided additional insight. When paxillin binding is abolished with the EKR/I937A mutant of FAK and focal adhesion localization is dramatically reduced, the ability of FAK to autophosphorylate on Y397 is not affected. Similarly, phosphorylation of Y861, which is a Src substrate, is not affected. However phosphorylation of Y576 and Y577, the Src phosphorylation sites in the activation loop of FAK, is impaired in this mutant. Several studies have indicated that the Y861 Src phosphorylation site on FAK is regulated differently than phosphorylation of the other Src sites on FAK, so this finding that Y576/Y577 and Y861 are differentially affected is not completely surprising [28-30]. Interestingly, mutants with defects in a single paxillin-binding site show similar defects in phosphorylation of Y576 and Y577, suggesting that engagement of both paxillin-binding sites is required for maximal phosphorylation of these sites. The fact that each of the paxillin-binding mutants show similar defects in phosphorylation of FAK indicates that phosphorylation of FAK at the activation loop sites does not precisely correlate with the ability of FAK to localize to focal adhesions. Paxillin binding to both sites on FAK may be required to allow Src binding to FAK, perhaps by tethering FAK to focal adhesions more securely. However, as Y397 phosphorylation is not altered, such a mechanism would indicate a defect in Src recruitment downstream of FAK autophosphorylation. A more likely scenario is that paxillin binding to both sites on FAK may cluster FAK proteins, thus facilitating phosphorylation of FAK by associated Src molecules.

A paxillin mutant defective for FAK binding is defective for tyrosine phosphorylation following adhesion and in *src*-transformed fibroblasts [18]. Thus it was anticipated that FAK mutants defective for paxillin binding would exhibit defects in their ability to promote paxillin phosphorylation. These mutants exhibited a parallel defect in p130CAS phosphorylation. The more severe defect in substrate phosphorylation observed in cells expressing the EKR/I937A mutant is likely due to the localization defect exhibited by the mutant resulting in the spatial segregation of the kinase and its substrate. The defect in p130CAS phosphorylation by I937A could partially reflect the decreased localization of the FAK mutant, but as the EKR mutant localizes correctly, its decreased ability to induce p130CAS phosphorylation cannot be attributed to a defect in localization. Instead, this defect correlates with reduced activation loop phosphorylation and may reflect reduced FAK activity and hence reduced substrate phosphorylation *in vivo*.

These findings provide interesting insight into how multiple binding sites for paxillin contribute to FAK function, but there is also a practical implication arising from these studies. Aberrant FAK signaling is associated with pathological conditions, e.g. the development of some cancers [31,32], and one experimental strategy to perturb FAK signaling is exogenous expression of the C-terminal non-catalytic domain, which functions as a dominant negative mutant [33-35]. More efficacious strategies to inhibit FAK signaling therapeutically would entail select inhibition of the interaction of FAK with critical binding partners. Our findings suggest that simultaneous disruption of both paxillin-binding sites would be required for maximal inhibition of FAK localization and function. Perhaps as importantly these findings also suggest that partial attenuation of FAK function might be obtained by selectively disrupting the interaction of paxillin with one of its two binding sites in the FAT domain. Such a strategy might allow attenuation of FAK signaling in target tissue without ablation of FAK signaling in normal tissue, which may reduce the deleterious effects of such therapeutic approaches.

## Conclusion

The main conclusions of this study are 1) a single functional paxillin-binding site can target FAK to focal adhesions, 2) both paxillin-binding sites are required to promote maximal phosphorylation of activation site tyrosines in FAK and 3) both binding sites are required for maximal phosphorylation of downstream substrates.

## Methods

### Molecular biology

FAK mutants were engineered using pBluescript-FAK or pEGFP-EKR/I937A as a template. Point mutations were engineered using the QuikChange mutagenesis kit (Stratagene, La Jolla CA). Sequence analysis was performed to verify the intended point mutations and that no unintended mutations were present. These analyses were performed in the UNC-CH Genome Analysis Facility on a model 3730 DNA Analyzer (Perkin Elmer, Applied Biosystems Division) using the ABI PRISM™ Dye Terminator Cycle Sequencing Ready Reaction Kit with AmpliTaq DNA Polymerase, FS (Perkin Elmer, Applied Biosystems Division). Full-length FAK cDNAs were subcloned into RCAS A as described previously [36]. Full-length FAK cDNAs were subcloned into pEGFP as described previously [37]. All subcloned constructs were verified by sequence analysis.

### Cells and viruses

Chicken embryo (CE) cells were prepared and maintained as described previously [38]. These cells were transfected using Lipofectamine PLUS (Life Technologies, Gaithersburg, MD) with RCAS A retroviral vectors encoding wild type or mutant FAK proteins [24]. To address the regulation of FAK phosphorylation in response to adhesion, cells were trypsinized, washed in 0.5 mg/mL soybean trypsin inhibitor (Sigma, St. Louis, MO) in PBS, held in suspension for 30 minutes, and replated onto dishes coated with fibronectin (50 μg/ml) for 45 minutes. In order to enhance the phosphotyrosine content of cellular proteins in some experiments, cells were treated with 50 μM sodium vanadate for 16 hrs prior to lysis [39].

CHO cells were maintained in DMEM containing 10% FBS, 4 mM L-glutamine, 1 mM sodium pyruvate and 1% non-essential amino acids. To generate stable populations of CHO cells expressing GFP tagged constructs, the cells were transfected with plasmids encoding wild type FAK or mutants fused to GFP using Lipofectamine PLUS (Life Technologies), followed by selection and maintenance in 1 mg/ml G418 (Gibco BRL). Populations of G418 selected CHO cells were then sorted by FACS to enrich for GFP expression. Trypsinized CHO cells were washed once in PBS then resuspended in 0.5 ml PBS. Flow cytometry was performed on a Becton Dickinson FACScan interfaced to a Cytomation, Inc. Cicero data acquisition system in the UNC Flow Cytometry Facility.

### Protein analysis

Cells were washed twice with PBS and lysed in modified RIPA buffer (50 mM Tris [pH 7.3], 150 mM NaCl, 1% Triton X-100, 0.5% deoxycholate) containing protease and phosphatase inhibitors. Protein concentrations were determined with the bicinchoninic acid assay (Pierce, Rockford, Ill.). The FAK phosphorylation site-specific antibodies (Biosource International, Camarillo, CA) and paxillin, p130CAS, and PY20 phosphotyrosine antibodies (BD Biosciences, San Diego, CA) were purchased commercially. The BC4 polyclonal antiserum has been described previously [40]. Immunoprecipitations and Western blotting were performed as previously described [26].

### Immunofluorescence

Glass coverslips were coated with 50 μg/mL bovine plasma fibronectin (Sigma) in PBS for 1 hr at 37°C. Cells were plated onto the coated coverslips and maintained at 37°C for 16 hr. Cells were fixed in 3.7% formaldehyde and permeabilized with 0.5% Triton X-100. FAK was detected using BC4 and rhodamine-conjugated anti-rabbit antibody (Jackson ImmunoResearch Labs). For co-staining studies, a paxillin monoclonal antibody (BD Biosciences) and rhodamine conjugated anti-mouse secondary antibodies (Jackson Labs) were used to visualize focal adhesions while an avian GFP antibody (Chemicon International, Temula, CA) and FITC anti-chicken secondary antibodies (Jackson Labs) were used to detect GFP-tagged proteins. Cells were visualized using a Leitz Orthoplan fluorescence microscope, and images captured with a Hamamatsu digital camera and Metamorph imaging software (Universal Imaging Corporation, West Chester, PA). Images were taken with identical exposure times. Paxillin staining was used to identify focal adhesions and cells were scored for co-localization of paxillin and GFP staining, indicating FAK localization at these sites. The results were expressed as percentage of cells exhibiting focal adhesion localization of GFP-FAK to focal adhesions. ANOVA analyses with Dunnett post-tests were performed using GraphPad software (San Diego, CA) to identify statistically significant differences in localization efficiency. For live cell imaging, EGFP N-terminally tagged forms of wild type FAK or the FAK mutants were expressed in CE cells using Lipofectamine PLUS (Invitrogen). At 24 hrs, the transfected cells were plated in 35 mm glass bottom dishes (MatTek, Ashland, MA) and incubated overnight at 37°C. At 48 hrs, cells were viewed using an Olympus IX81 microscope by epifluorescence and total internal reflection fluorescence (TIRF) microscopy.

### Protein preparation and *in vitro *binding experiments

GST fusion proteins were expressed in *E. coli *and purified as described [26]. Briefly, expression was induced with 0.1 mM isopropyl 1-thio-β-D-galactopyranoside, followed by incubation for 2 hrs at 37°C. The bacteria were harvested and sonicated in TETN buffer (1% Triton X-100, 20 mM Tris [pH 8.0], 100 mM NaCl, 1 mM EDTA) plus protease inhibitors (1 mM PMSF, 10 μg/mL leupeptin, 10 U/mL aprotinin). Clarified supernatants were bound to glutathione-agarose beads (Sigma) for 1 hr at 4°C, washed three times with PBS, and resuspended in equal volumes PBS. Fusion proteins were quantified by SDS/PAGE and Coomassie blue staining. For binding experiments, 1 mg of CE lysate was precleared by incubation with 100 μg of GST bound to glutathione-agarose beads for 1 hr at 4°C, then incubated with 25 μg GST fusion proteins immobilized on beads for 2 hrs at 4°C. The beads were washed twice with modified RIPA buffer, twice with PBS, eluted in sample buffer, and analyzed by western blotting.

## List of abbreviations used

FAK, focal adhesion kinase; 

FAT, focal adhesion targeting; 

LD, leucine aspartic acid; 

PI3K, phosphatidylinositol 3'-kinase; 

Grb, growth factor receptor binding protein; 

SH, src homology; Y, tyrosine; 

ERK, extracellular signal regulated kinase; 

CE, chicken embryo; 

CHO, Chinese hamster ovary; 

GFP, green fluorescent protein; 

FACS, fluorescence-activated cell sorting; 

MEF, mouse embryo fibroblast; 

TIRF, total internal reflection fluorescence; 

GST, glutathione-S-transferase.

## Competing interests

The author(s) declare that they have no competing interests.

## Authors' contributions

DS designed, performed the majority of the studies described and drafted the manuscript. JH designed and performed some studies. ZR assisted in the microscopy. GG and SC aided in the design of the study. MS conceived and designed the study and drafted the manuscript. All authors read and approved the final version of the manuscript.
